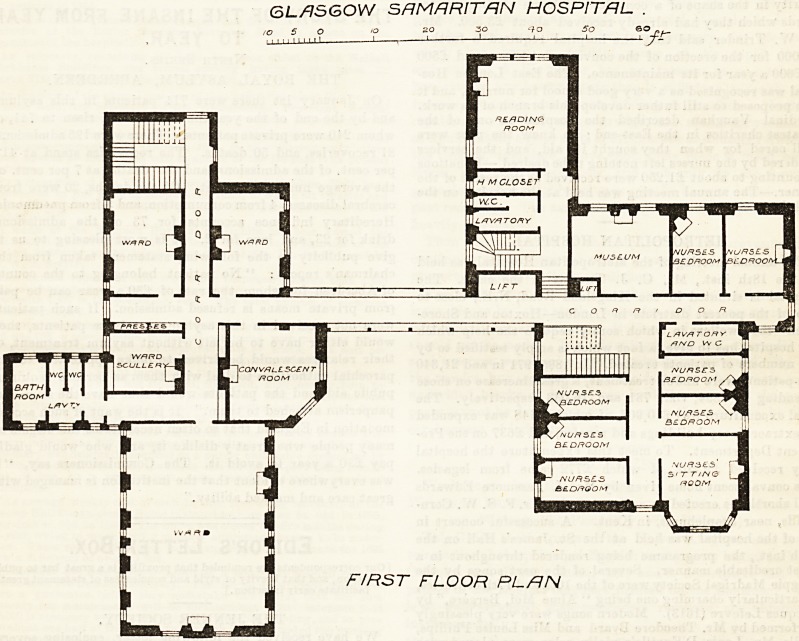# Hospital Construction

**Published:** 1896-05-23

**Authors:** 


### HOSPITAL CONSTRUCTION.

### GLASGOW SAMARITAN HOSPITAL FOR WOMEN.

The new buildings for this institution occupy an isolated site extending to about three-quarters of an acre, having a southern frontage to a piece of open ground reserved for a public square.

The plans, prepared by Messrs. McWhannel and Rogerson, show a building designed in two blocks—the hospital and administrative—connected by corridors five feet wide. The hospital block comprises two floors, each containing a ward for nine patients, two wards for two patients each, and a convalescent-room, a ward scullery, linen-room, and sanitary accommodation. The administrative block is three storeys in height, and includes certain rooms for educational work; on the ground floor are placed a waiting-room, board-room, matron's bed and sitting rooms, and surgeon's bed and sitting rooms, an operating-room, and instrument-room, as well as a lecture-room for forty students. On the first floor, in addition to bed-rooms and a sitting-room for nurses, a reading-room and museum are planned over the lecture-room, &c., and these are available for the use of students by a separate staircase, without entering.

GROUND FLOOR PLAN

the hospital block or the administrative portion of this block.

The second floor contains the kitchen, scullery and stores, &c., and sleeping-rooms for servants.

In the hospital block the large wards are 26 feet wide and warmed by two open fireplaces each. The position of the beds to be accommodated has obviously been well considered, and in this respect the small wards are also very well arranged. The large wards are, however, separated by a corridor more or less public both from the ward scullery and from the sanitary appliances, among which no sluice appears to have been provided. This has probably been done with a view to making the sanitary appliances available for all the wards and fully expedient, and the position of the H.M. closet with regard to the students' reading-room, &c., still more open to objection. No proper attention to the necessity of quietude for the rooms for night nurses or of bath-room accommodation for any of the nurses seems to have been exercised, and in these respects the plans appear capable of improvement, and some of the bed-rooms provided for the nurses are uncomfortably long in proportion to their width.

GLASGOW SAMARITAN HOSPITAL.

FIRST FLOOR PLAN

for the convalescent-room, but seems likely to prove in practice an awkward arrangement so far as the large wards are concerned. Another questionable arrangement in connection with what is really a portion of the patients' department is the remoteness of the operating room (placed in the administrative block) from the wards, and the want of any preparation room for patients previous to operation. The separation of the instrument-room from the operating-room by a public corridor is also unadvisable.

In the administrative block the sanitary arrangements for the students seem inadequate in proportion to the number provided for. On the first floor the position of the museum for students' use in regard to the nurses' bed-rooms is doubt-

## Figures and Tables

**Figure f1:**
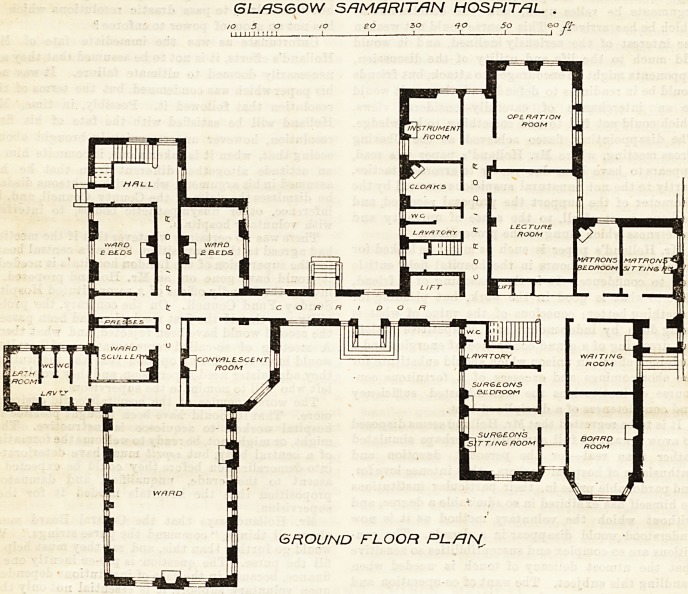


**Figure f2:**